# Prevalence of *kdr* mutations and insecticide susceptibility among natural population of *Aedes aegypti* in West Bengal

**DOI:** 10.1371/journal.pone.0215541

**Published:** 2019-04-15

**Authors:** Pabitra Saha, Moytrey Chatterjee, Sudeep Ballav, Akash Chowdhury, Nandita Basu, Ardhendu Kumar Maji

**Affiliations:** 1 Department of Microbiology, Calcutta School of Tropical Medicine, Kolkata, West Bengal, India; 2 Department of Zoology, A. P. C. Roy Govt. College, Himachal Bihar, Matigara, Siliguri, West Bengal, India; 3 Department of Pathology, Jagannath Gupta Institute of Medical Sciences and Hospital, Budge Budge, Kolkata, West Bengal, India; Centro de Pesquisas René Rachou, BRAZIL

## Abstract

**Background:**

*Aedes albopictus* and *Aedes aegypti* are the major vectors of arboviral diseases. As effective vaccines are not available for most of the arboviral diseases, vector control by using insecticides play the key role to reduce the disease transmission. The emergence and spread of resistance to different classes of insecticides by the vectors is a major obstacle to control the disease transmission. Information about vector susceptibility to different insecticides and their mechanisms are very important for formulating proper vector control measures. The present study was designed to assess the susceptibility of *Ae*. *aegypti* against three different classes of adulticides, one larvicidal agent available and polymorphisms in the voltage-gated sodium channel (*VGSC*) gene related to insecticide resistance.

**Methods:**

Immature stages of *Ae*. *aegypti* were collected from three dengue endemic municipal areas of West Bengal and reared in the laboratory. Larvae and adults (F1 progeny) were used for insecticide bioassay as per WHO protocols. Knock down resistance gene (*kdr*) mutations were assessed by direct sequencing of PCR products.

**Results:**

The *Ae*. *aegypti* population was found to be susceptible to type II pyrethroids and malathion but highly resistant to DDT. A high rate of polymorphisms in the *VGSC* gene was observed among the collected mosquitoes. A double mutant V1016**G** + F1534**C** was found to be associated with DDT resistance but neither V1016**G** nor F1534**C** alone showed the same association. Association between the *kdr* mutations and the susceptibility status of pyrethroids could not be established due to very small sample size. A low to moderate level of resistance was noticed against temephos among the larval population based on WHO criteria.

**Conclusion:**

The replacement of DDT by type II pyrethroids for the management of dengue vectors is an appropriate decision taken by the national program which is supported by the findings of a higher level of resistance to DDT. Persistence of polymorphisms in the *VGSC* gene might be an indication of emergence of resistance against pyrethroid insecticides that should be monitored at a regular interval. Attempts should be made to determine the effectiveness of other larvicides for replacement of temephos if needed in future. Along with the chemical insecticides different biological vector control methods as well as biopesticides should also be used in vector control programmes.

## Introduction

Mosquito-borne arboviral diseases like dengue, chikungunya, yellow fever and Zika are major public health problem with more than 4 million disability adjusted life years globally [1, 2]. The major causes behind emergence and spread of arboviral diseases are demographic changes, massive urbanization, population movement, trade, transport and lack of effective vector control strategies which favour the world-wide distribution of these viruses and vector mosquitoes [3, 4, 5, 6, 7]. During the last decade a higher level of mortality and morbidity has been observed due to dengue and Zika virus infection [8]. Both these diseases are mainly transmitted by *Aedes albopictus* and *Ae*. *aegypti* mosquitoes [9, 10]. The spread of the vectors was amplified during the Second World War due to rapid human movement and transportation leading to dengue epidemic [6]. After the war, rapid urbanization led to rapid spread of dengue and hyper-endemicity with multiple serotypes in most South East Asian countries, with severe forms of the disease [11]. Urban and sub-urban colonization comes with new man-made breeding sites for mosquitoes such as regular water containers, disposed water-holding vessels, waste disposal areas, small containers, and discarded tyres all that may help *Ae*. *albopictus* and *Ae*. *aegypti* to thrive and multiply [4, 12, 13]. *Ae*. *albopictus* and *Ae*. *aegypti* are potential vectors for dengue epidemics as they breed preferentially in artificial containers [14, 15, 16, 6]. To date no effective anti-viral agent is recommended against arboviruses including dengue virus. A vaccine against dengue, Dengvaxia^®^ (CYDTDV), has been licensed since 2015, but the overall efficacy of trials has been about 60% and it has not been used on a large scale [17]. Recently the World Health Organization (WHO) does not recommend wide spread vaccination with Dengvaxia^®^ as it increases the rate of dengue haemorrhagic fever in sero-negative individuals [18]. Effective vector control plays the key role for reducing transmission of arboviruses worldwide and is the essential component of the WHO strategy for the prevention, control, and elimination of Neglected Tropical Diseases [19]. However, the emergence and spread of insecticide resistance in vector mosquitoes is becoming a major obstacle to reaching the goals set by WHO. Resistance to different classes of insecticides have been recorded among both the *Aedes* vector species in different parts of the World [20]. The worldwide insecticide resistance network supported by the World Health Organisation is established to track insecticide resistance among the vectors of arboviruses and to evaluate the potential for deployment of alternative vector control interventions [21]. Four mechanisms have been found to be associated with insecticide resistance-metabolic enzyme-based resistance, reduced target site sensitivity due to mutations in target genes, reduced penetration of insecticide due to thickening of the cuticles and behavioural changes [22]. The first two mechanisms are studied extensively [23, 24, 25, 26] but the role of cuticular penetration has not been well explained [20]. Increased production of three metabolic enzymes i.e. cytochrome P450 monooxygenases (P450s), esterases and glutathione S-transferases are principally associated with insecticide resistance [22, 27]. Resistance due to target site insensitivity is associated with mutations at the *VGSC* gene, commonly referred to as knockdown resistance (*kdr*). The VGSC mutations modify the target site of insecticide so that insecticide does not bind and cause the prolonged opening of the sodium channel resulting in rapid paralysis of the insects [28].

In India, vector control measures against *Aedes* mosquitoes are primarily based on use of temephos as a larvicide, thermal fogging and ultra-low volume space spray of malathion to control dengue outbreaks and use of pyrethroid-treated bed nets to reduce human vector contact [29]. Until the recent past DDT was used as an indoor residual spray (IRS), that has been replaced by a synthetic pyrethroid (type II, alpha-cypermethrin). Several reports are available on insecticide resistance status of the dengue vector [30, 31, 32, 33, 34] from India, but such reports from West Bengal are very rare particularly for *Ae*. *aegypti* [35]. A regular monitoring of insecticide resistance and studies on mechanisms behind it are very important to detect the effectiveness of the used insecticides and newer ones against the prevailing vector population of any geographical region. The present study was undertaken to determine the insecticide susceptibility status of *Ae*. *aegypti* to three different classes of adulticides, one larvicidal agent and polymorphisms in *VGSC* gene to correlate with observed insecticide susceptibility status.

## Materials and methods

### Study areas and mosquito sampling

**Fig 1:**
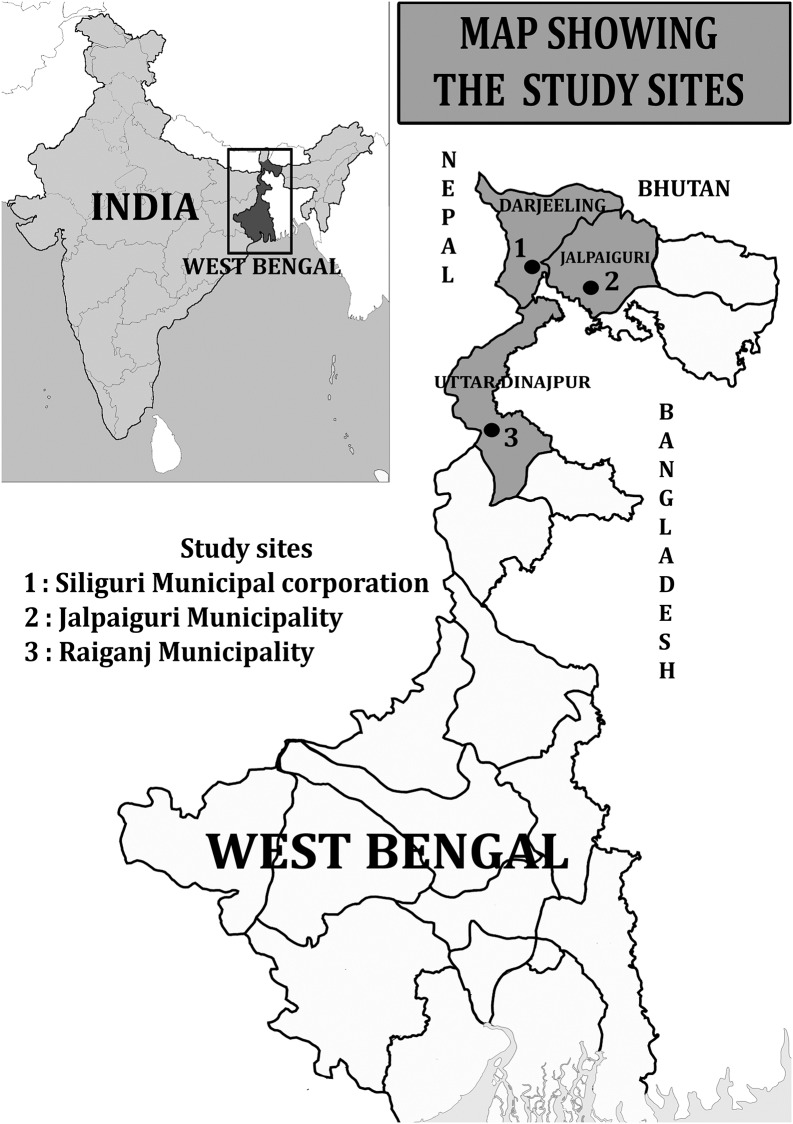
Map showing the study sites in three different districts of West Bengal.

The study was conducted in three different urban areas of West Bengal, namely, Siliguri Municipal Corporation (SMC) of Darjeeling (26.720695° N, 88.427686° E), Jalpaiguri Municipality of Jalpaiguri (26.544386° N 88.720568° E), and Raiganj Municipality of Uttar Dinajpur district (25.619691° N, 88.1256° E) (Fig 1). All the study sites were urban or sub-urban in nature. The study was under taken from March 2017 to June, 2018. The climatic conditions were humid and sub-tropical in nature and the temperature varies from 8°C in winter to 40° C in summer.

During the field survey, the study team visited house to house and looked for mosquito larvae and pupae in different natural breeding places present in and around the human dwellings. Different types of breeding sites like storage water tanks, discarded tyres, construction sites, flower pots, plastic cups, coconut shells, discarded containers etc were searched for *Aedes* sp. in all study areas. The larvae and pupae of *Aedes* sp. were collected from the domestic and peri-domestic natural breeding places. The collected immature stages of mosquitoes were gathered in plastic containers containing water from the breeding habitat and transferred to the laboratory.

### Mosquito rearing and identification

The collected larvae and pupae were transferred into a larva rearing tray in the laboratory and supplied with food for ornamental fishes and yeast available in the local market. The mosquito larvae and pupae were reared under controlled laboratory conditions such as temperature 25°C±2°C and humidity 80%±10%. The adult mosquito cages were supplied with suckling mice as a blood source for feeding the adult female mosquitoes. After emergence, the adult mosquitoes were identified using the standard identification keys of Barraud, 1934 [36] and Tyagi *et al*., 2012 [37]. The identified *Ae*. *aegypti* were allowed to breed under laboratory conditions. The larvae and adults of the F1 generation were used for insecticide bioassays.

### Larval susceptibility bioassay

The WHO standard bioassay protocol was used for estimation of susceptibility of *Ae*. *aegypti* larvae to temephos (procured from the Vector Control Research Unit, Universiti Sains Malaysia, Malaysia) [38]. Seven concentrations of temephos (0.001, 0.005, 0.01, 0.05, 0.1, 0.5 and 1.0 ppm) were prepared from 1 ppm stock temephos solution using 95% ethanol and used for larval bioassay as per WHO recommendations [39, 40]. Twenty to twenty-five late third instar to early fourth instar larvae were placed in each disposable paper cups filled with the required concentration of temephos solution and double distilled water at room temperature (25°C±2°C). Each set of bioassays was replicated at least four times and accompanied with two sets of controls (equal concentration of 95% ethanol). The mortality of larvae was recorded after 24 hours of exposure and was calculated by dividing the number of dead larvae by the total number of larvae tested. A test was considered invalid if pupation rate was greater than 10%, or mortality rate in the control was greater than 20% [38]. Field caught populations were reared for successive generations without any exposure to insecticides in the laboratory maintaining the controlled laboratory conditions mentioned earlier. The twentieth-generation larvae were used as a laboratory strain. The degree of resistance was determined by the resistance ratio (RR), which is calculated by comparing the lethal concentration (LC_50_) value for a study population with the LC_50_ value for a laboratory-maintained colony. When RR is <5 the field population is considered susceptible (S), when RR is between 5 and 10 mosquitoes are considered to have moderate resistance (MR), and when RR is >10 the mosquitoes are highly resistant (HR) [41].

### Adult susceptibility bioassay

The WHO adult bioassay protocol was used for determination of susceptibility status of adult *Ae*. *aegypti* against four different insecticides e.g.4% DDT, 0.05% deltamethrin, 0.05% alpha cypermethrin (Alpha-cyp) and 5% malathion [42]. Laboratory-emerged (F1 progeny), 2–3 days old unfed female *Ae*. *aegypti* mosquitoes were used for the bioassay. The adult bioassay kit and insecticide-impregnated papers were procured from the Vector Control Research Unit, Universiti Sains Malaysia, Malaysia. In each set of individual insecticide bioassays four experimental tubes (replicates) were set up and another one or two tubes were used as control. Before the experiment, 20–25 adult female mosquitoes were kept in each holding tube for one hour for acclimatization to experimental conditions. After acclimatization mosquitoes from four such tubes were exposed to insecticide-impregnated papers and one or two tubes to control tubes, respectively. Silicone oil was used as control for deltamethrin and alpha cypermethrin, olive oil, and risella oil for malathion and DDT respectively. Mosquitoes were exposed to insecticides for one hour and cumulative knock down was recorded after 10, 15, 20, 30, 40, 50, and 60 minutes. After exposure, the mosquitoes were transferred to holding tubes and fed on 5% sucrose solution for the next 24 hours. After that time, mortality was scored to determine the susceptibility status as per WHO recommendations [42]. Mosquitoes were considered dead if they were motionless, when they were mechanically stimulated, following the method of Gonzalez Audino [43]. The live and dead mosquitoes resulting from the bioassays were stored at -20°C and used for molecular assays.

### Data analysis

Larval bioassay data were analysed using Log dose probit (Ldp) Line computer software (Ehabsoft, Cairo Egypt; available at: http://www.ehabsoft.com/ldpline) according to Finney’s method [44]. Lethal concentrations (LC_10_, LC_50_, and LC_99_) along with the slope were estimated at 95% confidence intervals (CI). For adult bioassays, corrected mortality was calculated by using Abbott’s formula: Corrected Mortality (CM) (%) = [(% of observed mortality − % of control mortality) / (100 − % of control mortality)] x 100. Mosquitoes were considered susceptible (S) if the corrected mortality (CM) rate was greater than 98%; resistant (R) if mortality rate was less than 90% and mortality rate between 90–98% was considered as possible resistance (PR) and requiring verification by alternative methods like enzyme bioassay and molecular marker studies, as per WHO recommendation [42]. Knockdown time (KDT_10_, KDT_50_, and KDT_95_) is the time required for knockdown of a particular proportion of mosquitoes following exposure to any insecticide. KDTs were determined using Log dose probit (Ldp) Line computer software (Ehabsoft, Cairo Egypt; available at: http://www.ehabsoft.com/ldpline) programme according to the Finney’s method [44]. The association of point mutations with observed insecticide bio-assay was analysed by Fisher’s exact test using Graph pad (version 3.06).

### DNA isolation and *kdr* mutation detection

Genomic DNA was extracted from both live and dead mosquitoes (individually) by using the DNeasy Blood & Tissue Kit (Qiagen, Germany), as per the manufacturer’s instructions. Isolated DNA was stored at -20°C until further study.

PCR was done using two different primer pairs targeting important amino acid loci of domain II (S989**P**, I1011**M**, I1011**V**, V1016**G**, and V1016**I**) and F1534**C** of domain III of the *VGSC* gene, as described earlier by Kawada et al., 2016 [45]. PCR amplifications were carried out in a final volume of 50μl which include 3μl of genomic DNA as template. The reaction mixture contained PCR buffer, 0.2mM of dNTPs, 2.5 mM MgCl2, 0.3μM of each of the primer and 1.5U of AmpliTaq polymerase (Perkin Elmer, Branchburg, NJ, USA). The PCR reaction were carried out in Applied Biosystem Veriti96 well thermal cycler (Perkin Elmer, Branchburg, NJ, USA) and cycling parameters were an initial denaturation at 94° C for 3 minutes followed by 35 cycles of denaturation, 94° C for 15 s, annealing 55° C for 30 s and extension 72° C for 30s. The final elongation was done at 72° C for 10 min.

The quality of PCR products was ascertained by 2% agarose gel electrophoresis following ethidium bromide staining. The PCR product was gel purified using the Qiagen gel extraction kit (Qiagen, Germany) and sequencing was outsourced from Chromous Biotech, Bangalore. Two different primers AaSCR6 –CGACTTGATCCAGTTGGAGA (reverse primer for domain II) and AaSCR8– TAGCTTTCAGCGGCTTCTTC (reverse primer for domain III), as described earlier [44], were used for sequencing of the PCR products.

### Analysis of sequence

In the present study, we numbered the codon positions of *Ae*. *aegypti* (996, 1018, 1021, 1023 and 1565) corresponding to the positions of *Musca domestica* (989, 1011,1014,1016 and 1534 respectively) [23]. The sequences were analysed using the software BioEdit Sequence Alignment Editor version 7.0.9.0. The sequences were aligned with the reference sequence for *Ae*. *aegypti* (GenBank accession no. EU399181.1) using an online multiple sequence alignment (Pairwise sequence alignment) tool.

### Ethical statement

The aims and objectives of the study were explained to the local population of the study areas. Permission was taken from the owners of private houses/lands before collection of immature stages of mosquito. The study did not involve with any endangered and protected species. Mosquitoes were maintained under optimal conditions such as temperature, humidity, and adequate food supply in the laboratory. The study protocol was approved by the Institutional Ethics Committee of Calcutta School of Tropical Medicine, Kolkata.

## Results

### Larval susceptibility status

The results of larval susceptibility bioassay to temephos are presented in Table 1. The LC_50_ values of Siliguri MC, Jalpaiguri Municipality and Raiganj Municipality was 0.0168 mg/L, 0.0099 mg/L and 0.0079 mg/L, respectively; whereas LC_99_ values were 0.6684 mg/L, 0.3328 mg/L, and 0.3601 mg/L, respectively. The LC_50_ and LC_99_ values of laboratory strain were 0.0022mg/L and 0.0891mg/L, respectively. The calculated RR_50_ and RR_99_ values in Siliguri MC, Jalapiguri Municipality and Raiganj Municipality were 7.64, 7.5, 4.5 and 3.74, 3.59 and 4.04, respectively. So, the calculated RR_99_ values indicated that the *Ae*. *aegypti* larval population of Siliguri MC was moderately resistant (MR) to temephos, whereas larval population of Jalpaiguri Municipality and Raiganj Municipality were susceptible (S) to temephos.

**Table 1:** Temephos sensitivity status of *Ae*. *aegypti* larvae in West Bengal.

Values	Study sites			Laboratory/ susceptible strain
	Darjeeling	Jalpaiguri	Uttar Dinajpur	
	Siliguri MC (n = 320)	Jalpaiguri Municipality (n = 320)	Raiganj Municipality (n = 320)	
**LC**_**10**_ (95% CI) [mg/L]	0.0022 (0.0016–0.0029)	0.0014 (0.0006–0.0022)	0.001 (0.0003-.0.0014)	0.0002
**LC**_**50**_ (95% CI) [mg/L]	0.0168 (0.0139–0.0202)	0.0099 (0.0058–0.0157)	0.0079 (0.0038–0.0143)	0.0022
**LC**_**99**_ (95% CI) [mg/L]	0.6684 (0.4464–1.0996)	0.3328 (0.2032–1.0385)	0.3601 (0.2643–1.4563)	0.0891
**Χ**^**2**^ **(p)**	5.46 (0.36)	13.47 (0.019)	36.29 (<0.001)	371.42 (<0.0001)
**Slope**	1.45±0.08	1.52 ± 0.11	1.40 ± 0.07	1.45 ± 0.17
**R**	0.99	0.96	0.95	0.81
**G**	0.01	0.09	0.13	6.57
**RR**_**50**_**/RR**_**99**_	7.64/7.5	4.5/3.74	3.59/4.04	
**Status**^#^	**MR**	**S**	**S**	

**n** = number; **LC**_**10**_**/LC**_**50**_**/LC**_**99**_ = lethal concentration 10%/50%/99%, **RR** = resistance ratio, **g** = ‘g’ is a factor used for fiducial limit calculations

^**#**^Classification as per WHO, 2016: **S** = Susceptible (RR <5), **MR** = Moderate Resistance (5 <RR <10), **HR** = High Resistance (>10).

### Susceptibility status of adult *Ae*. *aegypti* to different insecticides

The results of the adult susceptibility bioassay are presented in Table 2. After 24 hours of exposure, the corrected mortality rates for 4% DDT were 68.20% to 74.70%. The obtained mortality rates were well below the WHO recommended 90% mortality rate for resistance. So, the results suggested that the *Ae*. *aegypti* population from the study areas were highly resistant to DDT. In all of the study sites, the corrected mortality rate for 0.05% deltamethrin was above 98%, except in SMC where the corrected mortality was 97.72%. The corrected mortality rates for 0.05% alpha cypermethrin and 5% malathion were >98.0% and >99.0% in all the study sites indicating that the natural population of *Ae*. *aegypti* of all the study areas were susceptible to deltamethrin, alpha cypermethrin and malathion except SMC where a low level of resistance was recorded to only deltamethrin.

**Table 2:** Insecticides susceptibility status of *Ae*. *aegypti* against 4% DDT, 0.05% deltamethrin, 5% malathion and 0.05% alpha cypermethrin in West Bengal.

Insecticides	Districts	Municipality	Mosquito exposed		Mosquito died		Observed Mortality (%)		CM (%)	KDT_10_ (Min) [95% CI]	KDT_50_ (Min) [95% CI]	KDT_95_ (Min) [95% CI]	χ^2^ (p)	Slope	Status#
			T*	C*	T*	C*	T*	C*							
**4% DDT**	Darjeeling	Siliguri MC	194	48	149	4	76.80	8.33	**74.70**	9.97 [8.22–11.58]	24.25 [22.23–26.33]	75.86 [64.89–92.98]	**4.09 (0.54)**	**3.32±0.25**	**R**
	Jalpaiguri	Jalpaiguri Municipality	168	44	117	2	69.64	4.55	**68.20**	11.83 [9.59–13.85]	32.54 [29.71–35.81]	119.21 [96.17–159.91]	**1.63 (0.89)**	**2.92±0.25**	**R**
	U. Dinajpur	Raiganj Municipality	185	45	134	2	72.43	4.44	**71.15**	10.44 [8.42–12.27]	28.15 [25.71–30.82]	100.61 [82.88–130.59]	**2.74 (0.74)**	**2.97±0.24**	**R**
**0.05% DEL**	Darjeeling	Siliguri MC	184	43	180	2	97.83	4.65	**97.72**	6.25 [4.83–7.55]	15.12 [13.47–16.66]	47.02 [40.85–56.56]	**10.25 (0.07)**	**3.34±0.28**	**PR**
	Jalpaiguri	Jalpaiguri Municipality	164	40	164	3	100.	7.50	**100.0**	5.76 [4.43–6.94]	12.28 [10.87–13.56]	32.52 [28.74–38.33]	**1.62 (0.89)**	**3.89±0.36**	**S**
	U. Dinajpur	Raiganj Municipality	172	48	169	3	98.26	6.25	**98.14**	7.41 [5.96–8.75]	17.65 [15.98–19.26]	53.76 [47.03–63.82]	**9.16 (0.10)**	**3.4±0.26**	**S**
**5% MAL**	Darjeeling	Siliguri MC	164	40	163	1	99.39	2.50	**99.37**	10.49 [7.98–11.98]	18.81 [15.86–21.68]	39.78 [34.9–50.9]	**15.99 (0.007)**	**5.05±0.33**	**S**
	Jalpaiguri	Jalpaiguri Municipality	170	42	170	2	100.0	4.76	**100.0**	7.24 [5.86–8.5]	16.01 [14.49–17.45]	44.28 [38.98–52.22]	**7.71 (0.17)**	**3.72±0.29**	**S**
	U. Dinajpur	Raiganj Municipality	160	41	160	1	100.0	2.44	**100.0**	10.01 [7.45–11.58]	19.32 [16.3–22.34]	44.94 [38.96–59.37]	**13.17 (0.02)**	**4.49±0.32**	**S**
**0.05% ALHA-CYP**	Darjeeling	Siliguri MC	162	40	160	1	98.77	2.5	**98.73**	6.10 [4.69–7.41]	14.89 [13.24–16.44]	46.84 [40.64–56.46]	**5.99 (0.31)**	**3.31±0.28**	**S**
	Jalpaiguri	Jalpaiguri Municipality	170	44	167	0	98.24	0	**98.24**	5.93 [4.62–7.16]	13.99 [12.43–15.44]	42.09 [37.21–49.24]	**2.38 (0.79)**	**3.44±0.27**	**S**
	U. Dinajpur	Raiganj Municipality	166	41	166	2	100.0	4.88	**100.0**	6.78 [5.45–8.01]	15.16 [13.66–16.56]	42.53 [37.67–49.65]	**8.38 (0.14)**	**3.67±0.28**	**S**

*T = Test, **C** = Control, **CM** = Corrected Mortality

^**#**^**S** = Susceptible (CM ≥98%); **R** = Confirmed Resistance (CM <90%); **PR** = Possible Resistance (CM = 90–97%)

**Fig 2:**
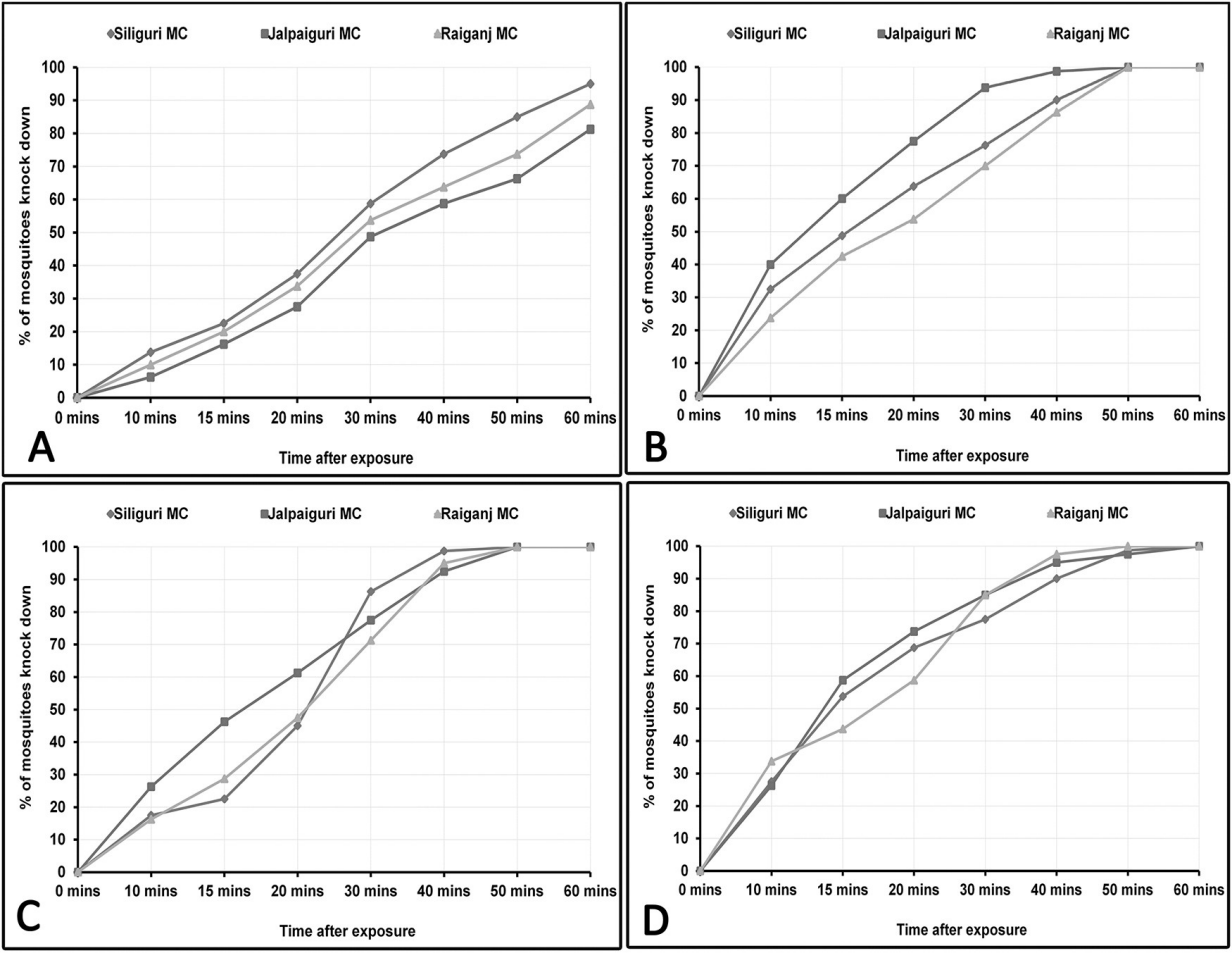
Knock down rate (KDR) of *Ae*. *aegypti* against 4% DDT. (A), 0.05% deltamethrin (B), 5% malathion (C), 0.05% alpha cypermethrin (D) in West Bengal.

The knockdown time (KDT_10_, KDT_50_, KDT_95_) for DDT, deltamethrin, alpha cypermethrin and malathion are given in Table 2. The observed KDT_50_ values were 24.25 to 32.54 mins for DDT, 12.28 to 17.65 mins for deltamethrin, 16.01 to 19.32 mins for malathion and 13.99 to 15.16 mins for alpha cypermethrin. The KDT_95_ values for DDT were 75.86 to 119.21 mins, for deltamethrin 32.52 to 53.76 mins for malathion, 39.78 to 44.94 mins and 42.09 to 46.84 mins for alpha cypermethrin. The knock down rate of *Ae*. *aegypti* against DDT, deltamethrin, malathion and alpha cypermethrin over an exposure time of 1 hour is given in Fig 2(A)–2(D).

### Prevalence of *kdr* mutations in *Ae*. *aegypti* and their association with insecticide resistance

*Kdr* mutations were successfully analysed among 110 mosquitoes, of which 46 were DDT exposed (alive 24, dead 22), 35 were deltamethrin exposed (alive 7, dead 28) and 29 were alpha cypermethrin exposed (alive 5, dead 24). An overall prevalence of V1016**G** (GTA→G**G**A) mutation was detected in 23 (20.91%, 95% CI: 14.36–29.43) samples, of which only 5 were heterozygous (V/**G**1016). Mutant F1534**C** (TTC→T**G**C) was detected in 58 (52.73%, 95% CI: 43.47–61.81) samples of which 9 were heterozygous (F/**C**1534) and 51 (46.36%, 95% CI: 37.32–55.64) samples harboured T1520**I** (ACC→A**T**C) mutation. We did not find heterozygous double mutant (V/**G**1016 + F/**C**1534) in any sample. A synonymous mutation in domain II, T1044T (ACT→AC**G**) was found in all the sample analysed. Two single mutant genotypes 1016**G** + 1534F and 1016V + 1534**C** were prevalent in 10.91% (95% CI: 6.35–18.11) and 42.73% (95% CI: 33.88–52.07) mosquitoes, whereas a double mutant genotype 1016**G** + 1534**C** was observed in 10% (95% CI: 5.68–17.02) mosquitoes (Table 3). The DNA sequences have been submitted to GenBank under accession numbers MK032480 and MK032481.

**Table 3:** Distribution of point mutations and combined genotypes of VGSC gene among *Ae*. *aegypti* exposed to DDT, deltamethrin and alpha-cypermethrin.

Phenotypes	N	Occurrence of point mutations n (%, 95% CI)						Occurrence of combined genotypes n (%, 95% CI)			
		1016		1520		1534		1016V+ 1534F	1016G+ 1534F	1016V+ 1534C	1016G+ 1534C
		Val (GTA)	Gly (GGA)	Thr (ACC)	Ile (ATC)	Phe (TTC)	Cys (TGC)				
DDT resistant (alive)	24	16 (66.67, 46.71–82.03)	8 (33.33, 17.97–53.29)	12 (50.0, 31.43–68.57)	12 (50.0, 31.43–68.57)	6 (25.0, 12.0–44.9)	18 (75.0, 55.1–88.0)	1 (4.16, 0.74–20.25)	5 (20.83, 9.24–40.47)	15 (62.5, 42.71–78.84)	3 (12.5, 4.34–31.0)
DDT sensitive (dead)	22	20 (90.91, 72.19–97.47)	2 (9.09, 2.53–27.81)	12 (54.55, 34.66–73.08)	10 (45.45, 26.92–65.34)	12 (54.55, 34.66–73.08)	10 (45.46, 26.92–65.34)	11 (50.0, 30.72–69.28)	1 (4.55, 0.81–21.8)	9 (40.9, 23.26–61.27)	1 (4.54, 0.81–21.8)
DEL resistant (alive)	7	3 (42.85, 15.82–74.96)	4 (57.15, 25.04–84.18)	4 (57.14, 25.04–84.18)	3 (42.86, 15.82–74.96)	2 (28.57, 8.22–64.11)	5 (71.43, 35.89–91.78)	1 (14.28, 2.57–51.32)	1 (14.29, 2.57–51.32)	2 (28.57, 8.22–64.11)	3 (42.85, 15.82–74.96)
DEL sensitive (dead)	28	25 (89.28, 72.81–96.29)	3 (10.72, 3.71–27.19)	18 (64.29, 45.83–79.3)	10 (35.71, 20.7–54.17)	18 (64.28, 45.83–79.3)	10 (35.72, 20.7–54.17)	16 (57.14, 39.07–73.49)	2 (7.14, 1.98–22.64)	9 32.14, 17.93–50.66)	1 (3.57, 0.63–17.71)
ALPHA-CYP resistant (alive)	5	2 (40.0, 11.76–76.93)	3 (60.0, 23.07–88.24)	2 (40.0, 11.76–76.93)	3 (60.0, 23.07–88.24)	3 (60.0, 23.07–88.24)	2 (40.0, 11.76–76.93)	1 (20.0, 3.62–62.45)	2 (40.0, 11.76–76.93)	1 (20.0, 3.62–62.45)	1 (20.0, 3.62–62.45)
ALPHA-CYP sensitive (dead)	24	21 (87.5, 69.0–95.66)	3 (12.5, 4.34–31.0)	11 (45.83, 27.89–64.92)	13 (54.17, 35.08–72.11)	11 (45.83, 27.89–64.92)	13 (54.17, 35.08–72.11)	10 (41.67, 24.47–61.17)	1 (4.17, 0.74–20.25)	11 (45.83, 27.89–64.92)	2 (8.33, 2.31–25.84)
**TOTAL**	**110**	**87** **(79.09, 70.57–85.64)**	**23 (20.91, 14.36–29.43)**	**59 (53.64, 44.36–62.67)**	**51 (46.36, 37.32–55.64)**	**52 (47.27, 38.19–56.53)**	**58 (52.73, 43.47–61.81)**	**40 (36.36, 27.97–45.67)**	**12 (10.91, 6.35–18.11)**	**47 (42.73–33.88–52.07)**	**11 (10.0, 5.68–17.02)**

Regarding distribution of genotype, single mutant 1016**G** + F1534 and V1016 + 1534**C** were recorded among five and fifteen DDT resistant (alive) mosquitoes & one and nine among DDT sensitive (dead) mosquitoes. By analysing with Fisher exact test, double mutant 1016**G** +1534**C** was found to be associated with DDT resistance (OR = 33.0, P = 0.0269), but no such association was recorded for individual point mutations at codon 1016**G** and 1534**C** (Table 3). As the number of tolerant mosquitoes obtained from adult bioassay with deltamethrin and alpha-cypermethrin were very few, we did not attempt to analyse any association of *kdr* mutation with them.

## Discussion

*Ae*. *aegypti* is highly anthropophilic, aggressive day biter with peak activities during early morning and late afternoon. They prefer to feed indoors and rest outside in close proximity to their breeding sites [16]. It is very difficult to control the adult mosquitoes through IRS (indoor residual spray) due to typical feeding and resting behaviour. Use of insecticides as space sprays using thermal fogging and ultra-low volume application are the choice of methods for controlling the *Aedes* population. In general, management of breeding sites with effective larvicides plays an important role for this purpose. The selection of effective insecticidal agents (larvicide and adulticide) is very important. In the present study, we attempted to determine the susceptibility status of *Ae*. *aegypti* in three different classes of insecticides; DDT (organochlorine), deltamethrin and alpha-cypermethrin (type II pyrethroid), malathion (organophosphate) as adulticide, temephos as larvicide and polymorphisms in *VGSC* gene among the mosquitoes collected from three different districts of West Bengal.

In India DDT was used as an insecticidal agent for a long time. The first case of DDT resistant *Ae*. *aegypti* was reported from India by Azeez (1967) [46] and then it spread widely across the country [32, 33,47,48,49]. In the present study we also observed a significantly high level of DDT resistance in all study sites with higher KDT and low KDR values. Alongside this problem, DDT has long term toxicity in the environment. So, the replacement of DDT by pyrethroid is an appropriate decision taken by the National Vector Borne Disease Control Programme (NVBDCP). Pyrethroid is a class of insecticide recommended by the World Health Organization for controlling mosquitoes due to its high efficacy against insects and low mammalian toxicity [50].

Emergence and spread of pyrethroid resistance in *Aedes* mosquitoes is a global problem for controlling vector borne arboviral diseases. A substantial geographical variation of pyrethroid resistance has been noted. Generally, a lower level of resistance was noticed in Asia and Africa whereas a higher level of resistance was observed in mosquitoes from the Caribbean, Mexico, and South America [51].

In the present study we observed that the *Ae*. *aegypti* populations of three study areas were susceptible to both the pyrethroids tested except in SMC of Darjeeling district where the corrected mortality was 97.72% for deltamethrin, slightly lower than the cut off level of 98% recommended by WHO [42]. A Similar observation was also made for *Ae*. *albopictus* of the same study site [52]. Though the pyrethroids have been introduced recently for control of vectors, the group has been used widely as a pesticide in agriculture. As all the study areas were urban in nature, exposure from agricultural applications of the insecticide was low and that might be the cause behind higher rate of pyrethroid sensitivity among the *Ae*. *aegypti* population. Unlike our study, pyrethroid resistance has been reported from different parts of India [33, 48] in contrary, pyrethroid sensitivity was also been reported [31, 47,49] from other parts of the country. *Ae*. *aegypti* from all of the study areas were highly susceptible to malathion. Similar observations have been made from different parts of the country [31, 47 and 48]. In the study areas malathion was used on rare occasions particularly to control dengue and Japanese Encephalitis outbreaks. If needed malathion might be an alternative to pyrethroid insecticides in future. Along with the chemical insecticides different biological vector control methods as well as biopesticides should also be used in vector control programmes.

The larval susceptibility of *Ae*. *aegypti* to temephos showed moderate resistance in one study area. In contrast temephos susceptibility was recorded from other parts of the country [31, 47,49]. The present study areas were urban and semi-urban in nature and controlled by local municipal authorities. The local authorities used temephos for management of mosquito breeding sites for a long time. This longer exposure might be the cause behind observed moderate resistance among the prevailing *Ae*. *aegypti* population of Siliguri MC area. So, the mode of use of temephos should be monitored closely or temephos should be replaced by other larvicidal agents if needed in future.

Several mutations in *VGSC* gene of *Ae*. *aegypti* have been reported, but only a few of them have been confirmed to be associated with pyrethroid resistance. In Asian countries, two *kdr* mutations V1016**G** and F1534**C** are common in *Ae*. *aegypti* [53, 54]. We also observed a high rate of point mutations at F1534**C** (52.73%) and V1016**G** (20.91%), similar to the observations from other parts of the country [32, 33].

There is a specific relation of these single nucleotide polymorphisms (SNPs) to the insecticide resistance. Mutant V1016**G** is reported to be associated with resistance to type I (permethrin) and type II (deltamethrin) pyrethroids, while F1534**C** with resistance to type I pyrethroids only [55]. In the present study mutant 1016**G** was not found to be associated with observed DDT resistance. Subsequently, the mutation at F1534**C** of S6 subunit of domain III was reported in DDT/ permethrin-resistant *Ae*. *aegypti* in Thailand and Vietnam [54, 56] but we did not observe any such association. Interestingly a double mutant (1016**G** + 1534**C**) in *VGSC* gene was found to be associated with resistance to DDT. But such correlation for deltamethrin and alpha-cypermethrin could not be established. Results may be confounded by a small sample size. We observed only five resistant mosquitoes of those that were exposed to alpha-cypermethrin and seven for deltamethrin.

A stepwise two additional mutations, S989**P** and D1763**Y** with V1016**G** were reported to be associated with permethrin resistant *Ae*. *aegypti* from south-east Asian countries [57, 58] but no such additional mutations were found in our study. An additive effect of double heterozygous mutation (V/G1016 + F/C1534) and triple heterozygous mutation (S/P989 + V/G1016 + F/C1534) to pyrethroid resistance have been reported from Thailand [59]. But no such double or triple heterozygous mutation was detected in the present study.

We observed a mutation at T1520**I** with a prevalence of (46.36%) which is similar to that observed by Khuswaha et al, 2015 [33] from India, but the role of this mutation in insecticide resistance is yet to be established.

## Conclusion

From the present study it was evident that the *Ae*. *aegypti* populations from each of the study areas were susceptible to the currently used pyrethroid i.e. 0.05% alpha cypermethrin and also to 0.05% deltamethrin, but highly resistant to DDT. However, presence of a high level of polymorphisms in *VGSC* gene may be an indication of emerging pyrethroid resistance. So, the susceptibility of used pyrethroid and polymorphisms in target genes should be monitored at regular intervals to detect the emergence of pyrethroid resistance among the *Ae*. *aegypti* population. As malathion is highly sensitive, it might be an alternative in near future if needed. A low to moderate level of resistance to temephos among larval populations was also noticed. Further study is required to observe the larval susceptibility to other larvicides to replace temephos for proper management of *Ae*. *aegypti*. Along with the chemical insecticides different biological vector control methods as well as biopesticides should also be used in vector control programmes.

## References

[1] Global Burden of Disease Study, 2013 Collaborators. Global, regional, and national incidence, prevalence, and years lived with disability for 301 acute and chronic diseases and injuries in 188 countries, 1990–2013: a systematic analysis for the Global Burden of Disease Study 2013. Lancet. 2015; 386:743–800. 10.1016/S0140-6736(15)60692-4 26063472 PMC4561509

[2] Naghavi M, Abajobir AA, Abbafati C, Abbas KM, Abd-Allah F, Abera SF, et al Global, regional, and national age-sex specific all-cause and cause-specific mortality for 240 causes of death, 1990–2013: a systematic analysis for the Global Burden of Disease Study 2013. Lancet. 2015; 385: 117–171. 10.1016/S0140-6736(14)61682-2 25530442 PMC4340604

[3] Corbel V, Fonseca DM, Weetman D, Pinto J, Achee NL, Chandre F, Coulibaly MB, Dusfour I, Grieco J, Juntarajumnong W, Lenhart A, Martins AJ, Moyes C, Ng LC, Raghavendra K, Vatandoost H, Vontas J, Muller P, Kasai S, Fouque F, Velayudhan R, Durot C, David JP. International workshop on insecticide resistance in vectors of arbovirus, December 2016, Rio de Janeiro, Brazil. Meeting report. Parasite & Vectors. 2017; 10:278. 10.1186/s13071-017-2224-3 PMC5457540 28577363

[4] Sutherst RW. Global change and human vulnerability to vector-borne diseases. Clin. Microbiol. Rev.2004;17(1):136–173. 10.1128/CMR.17.1.136-173.2004 14726459 PMC321469

[5] Gubler DJ. Dengue, Urbanization and Globalization: The Unholy Trinity of the 21(st) Century. Trop. Med. Health.2011;39(Suppl 4):3–11. 10.2149/tmh.2011-S05 PMC3317603 22500131

[6] Wilder-Smith A, Gubler DJ. Geographic expansion of dengue: the impact of international travel. Med Clin N Am.2008;92(6):1377–1390. 10.1016/j.mcna.2008.07.002 19061757

[7] Astrom C, Rocklov J, Hales S, Beguin A, Louis V, Sauerborn R. Potential distribution of dengue fever under scenarios of climate change and economic development. Eco. Health.2012;9(4):448–454. 10.1007/s10393-012-0808-0 23408100

[8] Global Burden of Disease Study 2015 Collaborators. Disease and Injury Incidence Prevalence Collaborators. Global, regional, and national incidence, prevalence, and years lived with disability for 310 diseases and injuries, 1990–2015: a systematic analysis for the Global Burden of Disease Study 2015. Lancet. 2016; 388:1545–602. 10.1016/S0140-6736(16)31678-6 27733282 PMC5055577

[9] Halstead SB. Dengue hemorrhagic fever–public health problem and a field for research. Bull. World. Health. Organ.1980; 58: 1–21. PMC2395896 6966540

[10] Gubler DJ. Dengue and dengue hemorrhagic fever. Clinic. Microbiol. Reviews. 1998;11: 480–496. 10.1128/cmr.11.3.480 PMC88892 9665979

[11] Gubler DJ, Clark GG. Dengue/dengue hemorrhagic fever: the emergence of a global health problem. Emerg. Infect. Dis. 1995; 1(2): 55–57. 10.3201/eid0102.952004 8903160 PMC2626838

[12] Mulligan K, Elliott SJ, Schuster-Wallace C. The place of health and the health of place: dengue fever and urban governance in Putrajaya, Malaysia. Health. Place.2012;18(3):613–620. 10.1016/j.healthplace.2012.01.001 22310527

[13] Mendonca HF, Ferreira AL, Santos CB, Rezende HR, Ferreira GEM, Leite GR, Falqueto A. Breeding sites of *Aedes aegypti* in metropolitan vacant lots in Greater Vitoria, State of Espirito Santo, Brazil. Rev. Soc. Bras. Med. Trop. 2011; 44(2):243–246. 21552743 10.1590/s0037-86822011000200022

[14] Padmanabha H, Durham D, Correa F, Diuk-Wasser M, Galvani A. The interactive roles of Aedes aegypti super-production and human density in dengue transmission. PLoS. Negl. Trop. Dis.2012;6(8):e1799 10.1371/journal.pntd.0001799 22953017 PMC3429384

[15] WHO Regional Office for South-East Asia. 2011 Comprehensive Guidelines for Prevention and Control of Dengue and Dengue Haemorrhagic Fever, Revised and Expanded Edition. New Delhi: World Health Organisation South East Asia Regional Office

[16] Morrison AC, Zielinski-Gutierrez E, Scott TW, Rosenberg R. Defining challenges and proposing solutions for control of the virus vector Aedes aegypti. PLoS. Med. 2008;5(3):e68 10.1371/journal.pmed.0050068 18351798 PMC2267811

[17] World Health Organization. 2017. Immunization, vaccines and biological. Geneva, Switzerland. http://www.who.int/immunization/research/development/dengue_q_and_a/en/

[18] World Health Organization. Dengue vaccine: WHO position paper–September 2018; Wkly Epidemiol Rec. 93:457–476. http://www.who.int/wer

[19] World Health Organization. 2012. Accelerating work to overcome the global impact of neglected tropical diseases—a roadmap for implementation. Geneva: World Health Organization; 2012 p. 22.

[20] Moyes CL, Vontas J, Martins AJ, Ng LC, Koou SY, Dusfour I, Raghavendra K, Pinto J, Corbel V, David JP, Weetman D. Contemporary status of insecticide resistance in the major Aedes vectors of arboviruses infecting humans. PLoS. Negl. Trop. Dis. 2017; 11(7): e0005625 10.1371/journal.pntd.0005625 28727779 PMC5518996

[21] Corbel V, Achee NL, Chandre F, Coulibaly MB, Dusfour I, Fonseca DM, Grieco J, Juntarajumnong W, Lenhart A, Martins AJ, Moyes C, Ng LC, Pinto J, Raghavendra K, Vatandoost H, Vontas J, Weetman D, Fouque F, Velayudhan R, David JP. Tracking Insecticide Resistance in Mosquito Vectors of Arboviruses: The Worldwide Insecticide Resistance Network (WIN). PLoS. Negl. Trop. Dis.2016;10(12): e0005054 10.1371/journal.pntd.0005054 27906961 PMC5131894

[22] Hemingway J, Ranson H. Insecticide resistance in insect vectors of human disease. Annu. Rev. Entomol. 2000; 45:371–91. 10.1146/annurev.ento.45.1.371 10761582

[23] Kasai S, Ng LC, Lam-Phua SG, Tang CS, Itokawa K, Komagata O, Kobayashi M, Tomita T. First detection of a putative knockdown resistance gene in major mosquito vector, *Aedes albopictus*. Jpn. J. Infect. Dis. 2011; 64: 217–221. 21617306

[24] Du Y, Nomura Y, Zhorov BS, Dong K. Sodium Channel Mutations and Pyrethroid Resistance in *Aedes aegypti*. Insects. 2016;7(4): 60. 10.3390/insects7040060 PMC5198208 27809228

[25] Liu Nannan. Insecticide Resistance in Mosquitoes: Impact, Mechanisms, and Research Directions. Annu. Rev. Entomol. 2015; 60:537–559. 10.1146/annurev-ento-010814-020828 25564745

[26] Faucon F, Dusfour I, Gaude T, Navratil V, Boyer F, Chandre F, Sirisopa P, Thanispong K, Juntarajumnong W, Poupardin R, Chareonviriyaphap T, Girod R, Corbel V, Reynaud S, David JP. Identifying genomic changes associated with insecticide resistance in the dengue mosquito *Aedes aegypti* by deep targeted sequencing. Genome. Res. 2015; 25(9):1347–59. 10.1101/gr.189225.115 26206155 PMC4561493

[27] Prapanthadara L, Reunkum W, Leelapat P, Suwan W, Yanola J, Somboon P. Glutathione S-transferase Isoenzymes and the DDTase Activity in Two DDT-resistant Strains of *Aedes aegypti*. Dengue Bulletin. 2005; 29:183–191.

[28] Hemingway J, Hawkes NJ, McCarroll L, Ranson H. The molecular basis of insecticide resistance in mosquitoes. Insect. Biochem. Mol. Biol. 2004;34(7): 653–665. 10.1016/j.ibmb.2004.03.018 15242706

[29] NVBDCP. 2015. Manual on Integrated vector management in India, http://nvbdcp.gov.in/WriteReadData/l892s/IVM-Manual-Draft-2015.pdf

[30] Dhiman S, Rabha B, Yadav K, Baruah I, Veer V. Insecticide susceptibility and dengue vector status of wild *Stegomyia albopicta* in a strategically important area of Assam, India. Parasite & Vectors. 2014; 7: 295. 10.1186/1756-3305-7-295 PMC4083132 24981885

[31] Yadav K, Rabha B, Dhiman S, Veer V. Multi-insecticide susceptibility evaluation of dengue vectors *Stegomyia albopicta* and *St*. *aegypti* in Assam, India. Parasite & Vectors. 2015; 8: 143 10.1186/s13071-015-0754-0 PMC4359396 25886449

[32] Muthusamy R, Shivakumar MS. Involvement of metabolic resistance and F1534C kdr mutation in the pyrethroid resistance mechanisms of *Aedes aegypti* in India. Acta. Tropica. 2015; 148: 137–141. 10.1016/j.actatropica.2015.04.026 25944353

[33] Kushwah RBS, Dykes CL, Kapoor N, Adak T, Singh OP. Pyrethroid-Resistance and Presence of Two Knockdown Resistance (kdr) Mutations, F1534C and a Novel Mutation T1520I, in Indian *Aedes aegypti*. PLoS. Negl. Trop. Dis. 2015(a);9(1): e3332. 25569164 10.1371/journal.pntd.0003332 PMC4287524

[34] Kushwah RBS, Mallick PK, Ravikumar H, Dev V, Kapoor N, Adak T, Singh OP. Status of DDT and pyrethroid resistance in Indian *Aedes albopictus* and absence of knockdown resistance (kdr) mutation. J. Vector. Borne. Dis. 2015(b);52: 95–98. 25815873

[35] Bharati M, Saha D. Assessment of Insecticide Resistance in Primary Dengue Vector, *Aedes aegypti* (Linn.) From Northern Districts of West Bengal. Acta tropica. 2018; 10.1016/j.actatropica.2018.07.004 (In press) 30026024

[36] Barraud PJ. The fauna of British India including Burma and Ceylon (Diptera: Culicidae), Tribes Megarhinini and Culicini, Vol 5, London: Taylor and Francis 1934;1–452

[37] Tyagi BK, Munirathinam A, Krishnamoorthi R, Venkatesh A. A field-based hand book of identification keys to mosquitoes of public health importance in India. Centre for research in Medical Entomology, Madurai, India 2012;25–27

[38] World Health Organization. 2005 Guidelines for laboratory and field testing of mosquito larvicides. WHO/CDS/WHOPES/GCDPP.13, Geneva, Switzerland: World Health Organization.

[39] World Health Organization. 1981(a). Criteria and meaning of tests for determining the susceptibility or resistance of insects to insecticides. WHO/VBC/81.806, Geneva.

[40] World Health Organization. 1981(b). Instructions for determining the susceptibility or resistance of adult mosquitoes to organochlorine, organophosphate and carbamate insecticides. Establishment of the baseline. WHO/VBC/81.805, Geneva.

[41] World Health Organization. Monitoring and managing insecticide resistance in Aedes mosquito populations Interim guidance for entomologists. 2016; http://apps.who.int/iris/bitstream/handle/10665/204588/WHO_ZIKV_VC_16.1_eng.pdf;jsessionid=453C3E558B3CF86802040C496E444171?sequence=2

[42] World Health Organization. 2016 Test procedures for insecticide resistance monitoring in malaria vector mosquitoes 2^nd^ ed World Health Organization, Geneva, Switzerland http://www.who.int/malaria/areas/vector_control/insecticide_resistance/en/

[43] Gonzalez AP, Vassena C, Barrios S, Zerba E, Picollo MI. Role of enhanced detoxication in a deltamethrin-resistant population of Triatoma infestans (Hemiptera, Reduviidae) from Argentina. Mem Inst. Oswaldo. Cruz. 2004;99(3): 335–339. doi: /S0074-02762004000300018 15273811 10.1590/s0074-02762004000300018

[44] Finney J. Probit analysis. 3rd En London, Cambridge University Press 1972;68–78.

[45] Kawada H, Higa Y, Futami K, Muranami Y, Kawashima E, Osei JHN, Sakyi KY, Dadzie S, Souza DK, Appawu M, Ohta N, Suzuki T, Minakawa N. Discovery of Point Mutations in the Voltage-Gated Sodium Channel from African *Aedes aegypti* Populations: Potential Phylogenetic Reasons for Gene Introgression. PLoS. Negl. Trop. Dis.2016;10(6): e0004780 10.1371/journal.pntd.0004780 27304430 PMC4909257

[46] Azeez SA. A note on the prevalence and susceptibility status of Aedes (Stegomyia) aegypti (Linn.) in Jharia, Dhanbad district (Bihar). Bull. Indian. Soc. Mal. Com. Dis. 1967;4: 59–62.

[47] Singh RK, Dhiman RC, Mittal PK, Dua VK. Susceptibility status of dengue vectors against various insecticides in Koderma (Jharkhand), India. J. Vector. Borne. Dis.2011; 48: 116–118. 21715737

[48] Marippan T, Selvam A, Rajamannar V, Arunachalam N. Susceptibility of Dengue/Chikungunya vector, *Aedes aegypti* against carbamate, organochlorine, organophosphate and pyrethroid insecticides. J. Environ. Biol. 2016; 30: 251–255.

[49] Katyal R, Tewari P, Rahman SJ, Pajni RH, Kumar K, Gill KS. Susceptibility Status of Immature and Adult Stages of *Aedes aegypti* Against Conventional Insecticides in Delhi, India. Dengue. Bulletin. 2001; 25: 84–87.

[50] Rehman H, Aziz AT, Saggu S, Abbas ZK, Mohan A, Ansari AA. Systematic review on pyrethroid toxicity with special reference to deltamethrin. J. Entomol. Zool. Stud. 2014;2(6): 60–70.

[51] Smith LB, Kasai S, Scott JG. Pyrethroid resistance in *Aedes aegypti* and *Aedes albopictus*: Important mosquito vectors of human diseases. Pestic. Biochem. Physiol. 2016; 133: 1–12. 10.1016/j.pestbp.2016.03.005 27742355

[52] Chatterjee M, Ballav S, Maji AK, Basu N, Sarkar BC, Saha P. Polymorphisms in voltage-gated sodium channel gene and susceptibility of *Aedes albopictus* to insecticides in three districts of northern West Bengal, India. PLoS. Negl. Trop. Dis.2018;12(1): e0006192 10.1371/journal.pntd.0006192 29309419 PMC5774824

[53] Brengues C, Hawkes NJ, Chandre F, McCarroll L, Duchon S, Guillet P, Manguin S, Morgan JC, Hemingway J. Pyrethroid and DDT cross-resistance in *Aedes aegypti* is correlated with novel mutations in the voltage-gated sodium channel gene. Med. Vet. Entomol. 2003;17(1):87–94. 12680930 10.1046/j.1365-2915.2003.00412.x

[54] Yanola J, Somboon P, Walton C, Nachaiwieng W, Somwang P, Prapanthadara L. High-throughput assays for detection of the F1534C mutation in the voltage-gated sodium channel gene in permethrin-resistant *Aedes aegypti* and the distribution of this mutation throughout Thailand. Trop. Med. Int. Health. 2011;16(4):501–509. 10.1111/j.1365-3156.2011.02725.x 21342372

[55] Stenhouse SA, Plernsub S, Yanola J, Lumjuan N, Dantrakool A, Choochote W, Somboon P. Detection of the V1016G mutation in the voltage-gated sodium channel gene of *Aedes aegypti* (Diptera: Culicidae) by allele-specific PCR assay, and its distribution and effect on deltamethrin resistance in Thailand. Parasite & Vectors. 2013; 6: 253. 10.1186/1756-3305-6-253 PMC3765916 24059267

[56] Kawada H, Higa Y, Komagata O, Kasai S, Tomita T, Thi Yen N, Loan LL, Sánchez RAP, Takagi M. Widespread Distribution of a Newly Found Point Mutation in Voltage-Gated Sodium Channel in Pyrethroid-Resistant *Aedes aegypti* Populations in Vietnam. PLoS. Negl. Trop. Dis.2009;3(10): e527 10.1371/journal.pntd.0000527 19806205 PMC2754656

[57] Srisawat R, Komalamisra N, Eshita Y, Zheng M, Ono K, Itoh TQ, Matsumoto A, Petmitr S, Rongsriyam Y. Point mutations in domain II of the voltage-gated sodium channel gene in deltamethrin-resistant *Aedes aegypti* (diptera: Culicidae). Appl. Entomol. Zool. 2010; 45: 275–282.

[58] Srisawat R, Komalamisra N, Apiwathnasorn C, Paeporn P, Roytrakul S, Rongsriyam Y, Eshita Y. Field-collected permethrin-resistant *Aedes aegypti* from central Thailand contain point mutations in the domainIIS6 of the sodium channel gene (kdr). Southeast Asian J. Trop. Med. Public Health.2012; 43: 1380–1386. 23413701

[59] Plernsub S, Saingamsook J, Yanola J, Lumjuan N, Tippawangkosol P, Sukontason K, Walton C, Somboon P. Additive effect of knockdown resistance mutations, S989P, V1016G and F1534C, in a heterozygous genotype conferring pyrethroid resistance in Aedes aegypti in Thailand. Parasites & Vectors, 2016; 9: 417. 27460671 10.1186/s13071-016-1713-0 PMC4962480

